# Insulin Resistance and Metabolic Syndrome Increase the Risk of Relapse For Fertility Preserving Treatment in Atypical Endometrial Hyperplasia and Early Endometrial Cancer Patients

**DOI:** 10.3389/fonc.2021.744689

**Published:** 2021-11-30

**Authors:** Xingchen Li, Yuan Fan, Jiaqi Wang, Rong Zhou, Li Tian, Yiqin Wang, Jianliu Wang

**Affiliations:** ^1^ Department of Obstetrics and Gynecology, Peking University People’s Hospital, Beijing, China; ^2^ Center of Reproductive Medicine, Peking University People’s Hospital, Beijing, China

**Keywords:** fertility-sparing treatment, atypical endometrial hyperplasia, insulin resistance, metabolic syndrome, recurrence

## Abstract

**Objective:**

Fertility-sparing treatment for young women with atypical endometrial hyperplasia (AEH) and early endometrial cancer (EC) is a difficult challenge. Insulin resistance (IR) and metabolic syndrome (MetS) are two potentially crucial, but currently enigmatic factors in the recurrence of AEH and early EC patients. In this study we attempt to elucidate these factors.

**Methods:**

A retrospective study was conducted from January 2010 to December 2019. Risk factors for recurrence and complete remission time after recurrence (RCR time) were investigated. ROC curves were built to estimate the accuracy of the metabolic characteristics and Kaplan–Meier (K–M) analysis was used to calculate recurrence-free survival (RFS) for patients with various IR or MetS statuses.

**Results:**

A total of 111 AEH or early EC patients met the criteria and were enrolled in our study. Univariate analysis found that BMI ≥25 kg/m^2^ (OR = 2.7, 95% CI: 1.1–6.4, *P* = 0.03), IR (OR = 9.5, 95% CI: 3.3–27.0, *P <*0.001), MetS (OR = 4.9, 95% CI:1.5–15.5, *P* = 0.008), IR+ and MetS+ (OR = 21.0, 95% CI: 4.8–92.7, *P <*0.001), histological type (OR = 3.5, 95% CI: 1.5–7.9, *P* = 0.003), and maintenance treatment (OR = 0.3, 95% CI: 0.1–0.6, *P* = 0.005) were all significantly associated with recurrence and longer RCR time. Among these factors, IR and MetS were determined to be two independent risk factors for recurrence. Moreover, using IR and MetS as markers significantly improved the diagnostic accuracy of recurrence for fertility-sparing treatment patients (AUC = 0.818, *P <*0.05) and may play synergistic roles in suppressing treatment. K–M analysis indicated both metabolic features played important roles in RFS (*P <*0.05).

**Conclusion:**

Both IR and MetS were significantly associated with recurrence and longer RCR time in AEH and early EC patients receiving fertility-sparing treatment.

## Introduction

Endometrial cancer (EC) is the most common gynecologic malignancy in developed countries (1), with an extensively increasing number of cases in China, particularly among younger women (2). Approximately 3–14% of EC cases are reported in premenopausal women under 40 who want to preserve their fertility (3). EC diagnosed in this age group is increasing in frequency and are typically early-stage, well-differentiated, endometrioid type adenocarcinomas (4). As the number of young EC patients rises, fertility-preserving therapy is becoming one of the most important conservative methods for these women and for the corresponding national policy in China. To date, progestin therapy has been the most common type of fertility-preserving therapy for atypical endometrial hyperplasia (AEH) and early EC (5).

Although the majority of patients show complete remission (CR) to conservative treatment, the recurrence rate is high, between 16.7 and 62%, and this probability continually increases with time (6). A systematic review and meta-analysis of risk factors for recurrence found that the recurrence rate at 1 and 2 years were 9.6% and 29.2%, respectively. However, this study failed to evaluate possible risk factors for the relapse of the disease (7).

Metabolic features including insulin resistance (IR) and metabolic syndrome (MetS) have long been regarded as some of the most essential risk factors for EC (8).

IR is defined as the reduced biological effect of a specified amount of insulin after binding to the receptor and is manifested as the decreased use and increased output of peripheral glucose (9). Previous study has demonstrated that IR occurs early during the development from hyperplasia to cancer in the endometrium (10). MetS, comprising obesity, dyslipidemia, hypertension, and hyperglycemia, is a cluster of risk factors not only for cardiovascular disease but some common cancers as well, notably EC (11, 12).

There are many studies that focusing on the relationship between IR/MetS and EC. However, it remains unclear whether IR and MetS have an impact on fertility-sparing treatment in AEHfocusing and early EC, especially in a recurrent event. In this study, we conducted a retrospective study to investigate the relationship between these metabolic features and recurrent events of preservative therapy for AEH and early EC patients. Furthermore, we explore the potential of IR and MetS in improving diagnostic accuracy for recurrence and prognostic prediction. Utilizing these markers may help us to further understand the role of metabolism in fertility-sparing treatment and better prevent recurrence in patients.

## Materials and Methods

### Patients

We retrospectively analyzed the data of patients who received fertility-sparing therapy for AEH and early (Grade 1, Stage IA) endometrioid adenocarcinoma (G1EA) from January 2012 to December 2019 in Peking University People’s Hospital. Baseline and clinicopathological data as well as follow-up data were collected.

Indications for conservative therapy for AEH and G1EA were as follows: (i) the patients were younger than 45 years and strongly desired to preserve their fertility; (ii) endometrial tissue sampling for diagnosis was carried out by dilation and curettage (D&C); and (iii) patients who were diagnosed with G1EA and underwent pelvic magnetic resonance imaging (MRI) for staging, myometrial invasion, or any displayed extra uterine lesions were ruled out by institutional radiologists. All patients agreed and signed informed consent for the treatment.

The patients were followed up with by July, 2020. The clinicopathological data from patients were retrieved from electronical medical records. This study was approved by the Ethics Committees of the Peking University People’s Hospital (No. 2020PHB063-01).

### Insulin Resistance and Metabolic Syndrome Evaluation

The homeostasis model assessment-insulin resistance (HOMA-IR) value was used to determine IR status. The HOMA-IR value was calculated as fasting blood glucose (FBG, mmol/L) × fasting insulin (FINS, μU/ml)/22.5. Patients with diabetes or whose HOMA-IR ≥2.95, were considered as insulin resistant (IR) (10). The MetS criteria were proposed by the Chinese Medical Association Diabetes Branch and defined as including three or four of the following criteria: 1) Overweight and/or obese, BMI (body mass index) is greater than 25.0 kg/m^2^. 2) High blood glucose, FBG is greater than 6.1 mmol/L and/or 2 h BG is greater than 7.8 mmol/L, and/or has been diagnosed with diabetes. 3) Hypertension, systolic/diastolic blood pressure was greater than 140/90 mmHg, and/or has been diagnosed with hypertension. 4) Dyslipidemia, blood TG is greater than 1.7 mmol/L, and/or fasting blood HDL <1.0 mmol/L (39 mg/dl).

### Treatment and Relapse

Patients were scheduled to receive 250/500 mg of medroxyprogesterone acetate (MPA), 160 mg of megestrol acetate (MA) orally, or GnRH on a daily basis for 12 weeks. After the treatment, endometrial biopsy was performed by D&C to assess the efficacy of the therapy after CR. CR was defined as a normal endometrium without atypical hyperplasia. The patients were followed up with every 3 to 6 months. Ultrasound and endometrial biopsy were used to evaluate the endometrium. Recurrence was defined as the reappearance of a lesion that had initially regressed following treatment. The relapse time studied was the time interval from CR to relapse during follow-up. Recurrence-free survival (RFS) was defined as the time, in months, from the date of achieving CR to the date of recurrence. RCR time was defined as the time required for CR after the primary recurrence.

### Statistical Analysis

Data are presented as mean ± SD or as counts with proportions. Possible risk factors associated with relapse or RCR time, including age, BMI, CA125, HOMA-IR, MetS, FBG, triglyceride, HDL, menstruation cycle, gestation, parity, family history (tumor history), hypertension, diabetes, Polycystic Ovarian Syndrome (PCOS), histological and progestin type, time to CR, and maintenance treatment after primary CR were investigated. Univariate and multivariate analyses were performed in the recurrence and non-recurrence groups using the mentioned risk factors by a logistic regression to determine the likelihood ratio and odds ratios (OR) were calculated along with 95% confidence intervals (95% CI). The rate of recurrence was analyzed using Kaplan–Meier (K–M) curve and compared between groups using a log-rank test. All of the analyses were performed with the statistical software packages of R version 3.4.3 (http://www.R-project.org, The R Foundation) and EmpowerStats (http://www.empowerstats.com, X&Y Solutions, Inc., Boston, MA). A two-sided significance level of <0.05 was considered statistically significant.

## Results

### Patients’ Selection and Characteristics

**Figure 1:**
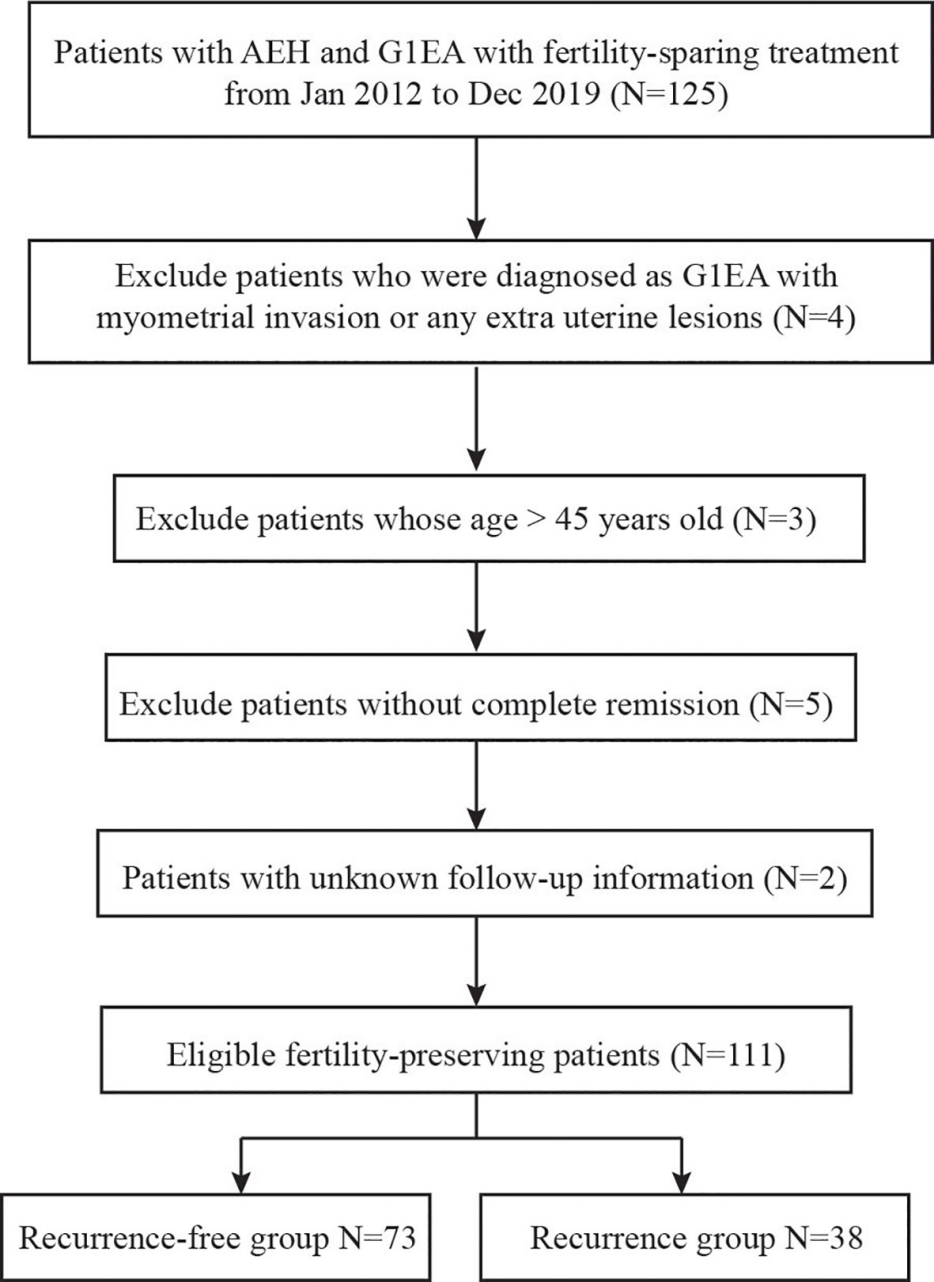
Flow diagram of inclusion criteria and exclusion criteria.

During the study period, a total of 63 EAH and 48 G1EA patients who met the inclusion criteria were evaluated (**Figure 1**). Baseline clinical characteristics between the recurrence-free and recurrence groups are shown in **Table 1**. The average age and BMI at diagnosis were 31.3 ± 4.5 years old and 26.6 ± 4.9 kg/m^2^, respectively. The mean value of HOMA-IR in the two groups was 3.5 and 3.9. The median length of RFS was 46.2 ± 32.1 months for the recurrence-free group, compared to 20.9 ± 17.5 months for the recurrence group. A total of 63 (56.8%) patients were diagnosed as IR, including 30 recurrence-free women and 33 recurrence women. Of the 101 patients, 15 (13.5%) had MetS and 10 out of 15 of these belonged to recurrent cases. The most common type of conservative therapy administered was MPA 250 mg (N = 59, 53.2%), followed by MPA 500 mg (N = 21, 18.9%), MA (N = 17, 15.3%), GnRH (N = 7, 6.3%), and combination therapy (N = 7, 6.3%). There were 40.5% patients (N = 45) who took less than 3 months to become CR, 32.4% (N = 36) took 3–6 months, and the rest (N = 30, 27.1%) took longer than 6 months. The majority of patients underwent maintenance treatment (N = 55, 65.4%, in the recurrence-free group and 55.3%, N = 21 in the recurrence group).

**Table 1:** Clinicopathological characteristics and treatment in recurrence-free and recurrence group.

Recurrence	Total	Recurrence	
	Mean+SD	No (N=73)	Yes (N=38)
Age at diagnosis (year)	31.3 ± 4.5	31.5 ± 4.5	31.1 ± 4.6
BMI (kg/m2)	26.6 ± 4.9	26.5 ± 5.0	26.9 ± 4.6
CA125 (U/mL)	22.5 ± 21.1	20.8 ± 21.7	25.7 ± 19.5
HOMA-IR	3.6 ± 2.7	3.5 ± 3.2	3.9 ± 1.7
Glucose (mmol/L)	5.3 ± 3.5	5.4 ± 4.2	5.1 ± 1.1
Triglyceride (mmol/L)	1.6 ± 1.1	1.6 ± 1.1	1.6 ± 1.0
HDL (mmol/L)	1.2 ± 0.4	1.2 ± 0.4	1.1 ± 0.3
RCR time (month)	3.8 ± 4.3	NA	7.3 ± 4.3
RFS (month)	37.5 ± 30.4	46.2 ± 32.1	20.9 ± 17.5
IR	N (%)	N (%)	N (%)
No	48 (43.2%)	43 (58.9%)	5 (13.2%)
Yes	63 (56.8%)	30 (41.1%)	33 (86.8%)
MetS			
No	96 (86.5%)	68 (93.2%)	28 (73.7%)
Yes	15 (13.5%)	5 (6.8%)	10 (26.3%)
Menstruation cycle			
Regular	56 (50.5%)	38 (52.1%)	18 (47.4%)
Irregular	55 (49.5%)	35 (47.9%)	20 (52.6%)
Gestation			
No	63 (56.8%)	36 (49.3%)	27 (71.1%)
Yes	48 (43.2%)	37 (50.7%)	11 (28.9%)
Parity			
No	92 (82.9%)	57 (78.1%)	35 (92.1%)
Yes	19 (17.1%)	16 (21.9%)	3 (7.9%)
Family history			
No	98 (88.3%)	67 (91.8%)	31 (81.6%)
Yes	13 (11.7%)	6 (8.2%)	7 (18.4%)
Hypertension			
No	103 (92.8%)	69 (94.5%)	34 (89.5%)
Yes	8 (7.2%)	4 (5.5%)	4 (10.5%)
Diabetes			
No	87 (78.4%)	60 (82.2%)	27 (71.1%)
Yes	24 (21.6%)	13 (17.8%)	11 (28.9%)
PCOS			
No	60 (54.1%)	37 (50.7%)	23 (60.5%)
Yes	51 (45.9%)	36 (49.3%)	15 (39.5%)
Histological type			
AEH	63 (56.8%)	49 (67.1%)	14 (36.8%)
G1EA	48 (43.2%)	24 (32.9%)	24 (63.2%)
Progestin type			
MPA250mg	59 (53.2%)	36 (49.3%)	23 (60.5%)
MPA500mg	21 (18.9%)	19 (26.0%)	2 (5.3%)
MA	17 (15.3%)	8 (11.0%)	9 (23.7%)
GnRH	7 (6.3%)	6 (8.2%)	1 (2.6%)
≥2 kinds	7 (6.3%)	4 (5.5%)	3 (7.9%)
Time to CR (month)			
<3	45 (40.5%)	31 (42.5%)	14 (36.8%)
3-6	36 (32.4%)	20 (27.4%)	16 (42.1%)
>6	30 (27.1%)	22 (30.1%)	8 (21.1%)
Maintenance treatment after primary CR			
No	35 (31.5%)	18 (34.6%)	17 (44.7%)
Yes	76 (68.5%)	55 (65.4%)	21 (55.3%)

SD, standard deviation; BMI, body mass index; HOMA-IR, homeostasis model assessment of insulin resistance-insulin resistance; HDL, high-density lipoprotein; RCR time, complete remission time after recurrence; RFS, recurrence-free survival; IR, insulin resistance; MetS, metabolic syndrome; PCOS, polycystic ovarian syndrome; AEH, atypical endometrial hyperplasia; CR, complete remission.

### Factors Associated With Recurrence and RCR Time

To investigate the relationship between clinicopahological indexes and recurrence, as well as explore the risk factors, a logistic regression analysis was conducted. Univariate analysis (**Table 2**) showed that a BMI ≥25 kg/m^2^ (OR = 2.7, 95% CI: 1.1–6.4, *P* = 0.03), IR (OR = 9.5, 95% CI: 3.3–27.0, *P <*0.001), MetS (OR = 4.9, 95% CI:1.5–15.5, *P* = 0.008), IR+ and MetS+ (OR = 21.0, 95% CI: 4.8–92.7, *P <*0.001), histological type (OR = 3.5, 95% CI: 1.5–7.9, *P* = 0.003), and maintenance treatment (OR = 0.3, 95% CI: 0.1–0.6, *P* = 0.005) appeared to be positively associated with recurrence. Upon further investigation it was demonstrated that BMI ≥25 kg/m^2^ (OR = 2.3, 95% CI: 1.7–3.9, *P* = 0.006), IR (OR = 3.0, 95% CI: 1.5–4.5, *P <*0.001), MetS (OR = 6.2, 95% CI: 4.2–8.2, *P <*0.001), IR+ and MetS+ (OR = 7.6, 95% CI: 5.5–9.8, *P <*0.001), histological type (OR = 2.5, 95% CI: 1.5–4.0, *P* = 0.002), and maintenance treatment (OR = 0.4, 95% CI: 0.1–0.7, *P* = 0.016) were also significantly related with recurrence. Unlike with recurrence, diabetes (OR = 2.8, 95% CI: 1.1–4.7, *P* = 0.004) was found to be a risk factor for RCR time. No relationships were found between other factors and recurrence or RCR time (*P >*0.05).

**Table 2:** Univariate analysis between clinicopathological characteristics and recurrence/RCR time.

	Recurrence		RCR time	
	OR (95%CI)	*P*	OR (95%CI)	*P*
Age (year)	1.0 (0.9, 1.1)	0.689	-0.1 (-0.2, 0.1)	0.510
BMI (kg/m2)	1.0 (0.9, 1.1)	0.679	0.1 (-0.1, 0.3)	0.220
CA125 (U/mL)	1.0 (1.0, 1.0)	0.266	0.0 (-0.0, 0.0)	0.674
HOMA-IR	1.1 (0.9, 1.2)	0.444	0.2 (-0.1, 0.5)	0.283
FBG (mmol/L)	1.0 (0.8, 1.1)	0.691	-0.0 (-0.3, 0.2)	0.799
Triglyceride (mmol/L)	1.0 (0.7, 1.4)	0.972	-0.0 (-0.8, 0.7)	0.914
HDL (mmol/L)	1.0 (0.1, 1.8)	0.270	-1.8 (-4.1, 0.5)	0.138
BMI				
<25kg/m^2^	1.0		1.0	
≥25kg/m^2^	2.7 (1.1, 6.4)	0.029	2.3 (1.7, 3.9)	0.006
IR				
Yes	1.0		1.0	
No	9.5 (3.3, 27.0)	<0.001	3.0 (1.5, 4.5)	<0.001
MetS				
Yes	1.0		1.0	
No	4.9 (1.5, 15.5)	0.008	6.2 (4.2, 8.2)	<0.001
IR+ and MetS+				
IR- and MetS-	1.0		1.0	
IR+ or MetS+	7.2 (2.4,21.1)	<0.001	1.6 (0.2, 3.0)	0.029
IR+ and MetS+	21.0 (4.8.92.7)	<0.001	7.6 (5.5, 9.8)	<0.001
Menstruation cycle				
Regular	1.0		1.0	
Irregular	1.2 (0.6, 2.6)	0.640	1.1 (-0.5, 2.7)	0.189
Gestation				
No	1.0		1.0	
Yes	0.4 (0.2, 0.9)	0.030	-1.4 (-3.0, 0.2)	0.083
Parity				
No	1.0		1.0	
Yes	0.3 (0.1, 1.1)	0.074	-1.7 (-3.8, 0.4)	0.107
Family history				
No	1.0		1.0	
Yes	2.2 (0.6, 7.2)	0.212	0.5 (-2.0, 3.1)	0.688
Hypertension				
No	1.0		1.0	
Yes	2.0 (0.5, 8.6)	0.337	1.9 (-1.2, 5.0)	0.231
Diabetes				
No	1.0		1.0	
Yes	1.9 (0.7, 4.7)	0.180	2.8 (1.1, 4.7)	0.004
PCOS				
No	1.0		1.0	
Yes	0.7 (0.3, 1.5)	0.325	-1.2 (-2.8, 0.4)	0.152
Histological type				
AEH	1.0		1.0	
G1EA	3.5 (1.5, 7.9)	0.003	2.5 (1.5, 4.0)	0.002
Progestin type				
MPA250mg	1.0		1.0	
MPA500mg	1.0 (0.3, 3.5)	0.967	-1.2 (-3.7, 1.3)	0.347
MA	2.5 (0.8, 7.3)	0.103	0.4 (-1.8, 2.7)	0.717
GnRH	0.4 (0.0, 3.2)	0.365	-2.1 (-5.4, 1.2)	0.217
≥2 types	1.6 (0.3, 8.0)	0.539	2.9 (-0.4, 6.2)	0.088
Time to CR (month)				
<3	1.0		1.0	
3-6	1.1 (0.6, 2.0)	0.621	0.8 (0.5, 1.5)	0.518
>6	2.0 (0.8, 5.1)	0.375	0.9 (0.4, 1.8)	0.857
Maintenance treatment after primary CR				
No	1.0		1.0	
Yes	0.3 (0.1, 0.6)	0.005	0.4 (0.1, 0.7)	0.016

SD, standard deviation; BMI, body mass index; HOMA-IR, homeostasis model assessment of insulin resistance-insulin resistance; HDL, high-density lipoprotein; RCR time, complete remission time after recurrence; RFS, recurrence-free survival; IR, insulin resistance; MetS, metabolic syndrome; PCOS, polycystic ovarian syndrome; AEH, atypical endometrial hyperplasia; CR, complete remission.

### Metabolic Features Were Independent Risk Factors for a Recurrent Event

To investigate the relationship between metabolic features and recurrence, multivariate analysis was conducted. Different models adjusting for different confounding factors were built to see whether IR and MetS were independent risk factors for the recurrence of fertility-sparing patients. Model I adjusted for baseline information including patient age, BMI, gestation, and parity. Model II added all significant risk factors in univariate analysis based on Model I, such as diabetes, histological type, and maintenance treatment. In the recurrence group, IR was found to be significantly associated with recurrence, whether confounding factors were adjusted for or not (Model I: OR = 13.3, 95% CI: 4.0–43.9, *P <*0.001; Model II: OR = 12.6, 95% CI: 3.7–43.3. *P <*0.001, **Table 3**), and MetS was also an independent risk factor for recurrence (Model I: OR = 5.5, 95% CI: 1.5–19.9, *P =* 0.009; Model II: OR = 5.8, 95% CI: 1.5–22.7, *P* = 0.012). In the RCR group (**Table 4**), both IR (Model I: OR = 2.9, 95% CI: 1.2–4.6, *P* = 0.002; Model II: OR = 2.6, 95% CI: 1.8–4.4, *P* = 0.005) and MetS (Model I: OR = 6.9, 95% CI: 4.7–9.0, *P <*0.001; Model II: OR = 6.8, 95% CI: 4.7–8.9, *P* = 0.005) were found to increase the risk of lengthened CR time after recurrence. IR and MetS were two of the major metabolic features for patients with both diagnoses, and patients with both diagnoses were more likely to have recurrence after CR (Model I: OR = 29.6, 95% CI: 17.5–388.0, *P <*0.001; Model II: OR = 45.6, 95% CI: 21.5–595.2, *P <*0.001), as well as a longer RCR time (Model I: OR = 8.2, 95% CI: 15.8–10.7, *P <*0.001; Model II: OR = 7.9, 95% CI: 5.4–10.3, *P <*0.001). Above all, IR and MetS were identified as two independent risk factors for both recurrence and RCR time in fertility-preserving patients, with the combination of both diagnoses intensively increasing risk.

**Table 3:** Logistic regression models evaluating the relationship between metabolic features and recurrence .

Exposure	Non-adjusted	Model I (OR, 95%CI) *P*	Model II (OR, 95%CI) *P*
IR			
No	1.0	1.0	1.0
Yes	9.5 (3.3, 27.0) <0.001	13.3 (4.0, 43.9) <0.001	12.6 (3.7, 43.3) <0.001
MetS			
No	1.0	1.0	1.0
Yes	4.9 (1.5, 15.5) 0.008	5.5 (1.5, 19.9) 0.009	5.8 (1.5, 22.7) 0.012
IR and MetS			
IR- and MetS-	1.0	1.0	1.0
IR+ or MetS+	7.2 (2.4,21.1) <0.001	16.1 (3.4,76.1) <0.001	19.1 (3.2, 115.4) <0.001
IR+ and MetS+	21.0 (4.8.92.7) <0.001	29.6 (17.5, 388.0) <0.001	45.6 (21.5,595.2)<0.001

Non-adjusted model adjusted model for none; Model I adjusted for: age, BMI; Model II adjusted for: age, BMI; IR; MetS; histological type; maintenance treatment.

IR, insulin resistance; MetS, metabolic syndrome; OR, odds ratio; CI, confidence interval.

**Table 4:** Logistic regression models evaluating the relationship between metabolic features and RCR time.

Exposure	Non-adjusted	Model I (OR, 95%CI) *P*	Model II (OR, 95%CI) *P*	
IR				
No	0	0		0
Yes	3.0 (1.5, 4.5) <0.001	2.9 (1.2, 4.6) 0.002		2.6 (1.8, 4.4) 0.005
MS				
No	0	0		0
Yes	6.2 (4.2, 8.2) <0.001	6.9 (4.7, 9.0) <0.001		6.8 (4.7, 8.9) <0.001
IR and MetS				
IR- and MetS-	0	0		0
IR+ or MetS+	1.6 (0.2, 3.0) 0.029	1.5 (-0.1, 3.2) 0.062		1.2 (-0.5, 2.8) 0.167
IR+ and MetS+	7.6 (5.5, 9.8) <0.001	8.2 (5.8, 10.7) <0.001		7.9 (5.4, 10.3) <0.001

Non-adjusted model adjusted model for none; Model I adjusted for: age, BMI; Model II adjusted for: age, BMI; IR; MetS; diatebes; histological type; maintenance treatment.

IR, insulin resistance; MetS, metabolic syndrome; OR, odds ratio; CI, confidence interval.

### IR and MetS Increase the Diagnostic Accuracy for Recurrence in AEH and G1EA Patients

**Figure 2:**
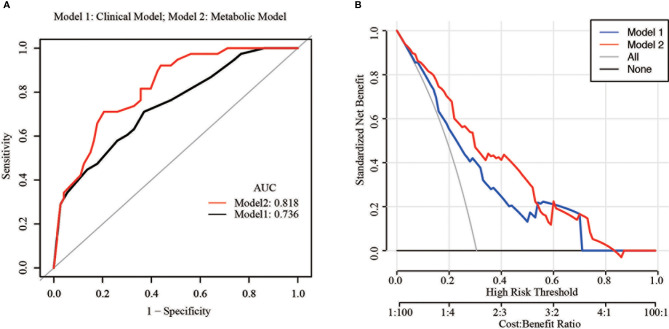
Predictive accuracy of different models. **(A)** Area under the receiver operating characteristic curves (AUCs) of models 1 and 2 for the prediction of recurrence in fertility-sparing treatment patients; **(B)** Decision curve analysis of the models.

In order to estimate the accuracy of metabolic features in predicting the recurrence in conservative EC patients, a ROC curve for different combinations of risk factors was built. The area under the ROC curve (AUC) ranges from 0 to 1 and a model is considered to have a poor, fair, or good performance if the AUC lies between 0.5–0.6, 0.6–0.7, and >0.7, respectively. As shown in **Figure 2A**, the AUC of the combination of three risk factors (clinical model) namely, BMI, histological type, and maintenance time was 0.74 (95% CI: 0.66–0.83). However, after adding in the two metabolic features, IR, and MetS (metabolic model), the AUC reached to 0.82 (95% CI: 0.70–0.89), a significant improvement over the clinical model (*P* = 0.034). The recurrence rates were 15.9 and 20.0% in metabolic model and clinical model, respectively (**Table S1**). The decision curve analysis (DCA) resulted for the clinical model and metabolic model are shown in **Figure 2B**. For predicted probability thresholds between 0% and nearly 60%, the metabolic model showed a positive net benefit for both the conservative treatment patients. Therefore, these results suggested that metabolic features significantly increased the accuracy in predicting recurrence of the fertility-sparing treatment.

### Prognostic Value of the IR and MetS for Relapse

To evaluate and test whether the metabolic features affected RFS in AEH and G1EA patients, K–M recurrence curve of different IR and MetS groups was conducted. Log-rank test analysis revealed that there was a significant difference in recurrence rate between IR group and the combination group (IR+ and MetS+ group). The median time for patients with or without IR was 20.9 and 46.2 months, respectively. K–M recurrence curve is shown in **Figure S1** and log-rank analysis revealed that the recurrence rate of women with IR was significantly lower than that of those without IR (P <0.05, **Figure S1A**). Patients were then divided into three groups according to their metabolic characteristics: IR− and MetS−, IR+ or MetS+, and IR+ and MetS+. K–M recurrence curve demonstrated that the recurrence time frame of women with IR+ and MetS+ was significantly lower than that in the other two groups (P <0.05, **Figure S1C**). However, no difference in recurrence speed was found between MetS+ and MetS− groups (P >0.05, **Figure S1B**). These K–M curves revealed that IR, especially combined with MetS, has a great influence on recurrence in fertility-sparing treatment patients.

## Discussion

Considering the rising incidence of endometrial cancer (EC) in reproductive age women and their relatively good prognosis, it is imperative to provide them with an effective fertility-preserving treatment option. Especially in China, where such work could actively address the country’s aging trend by facilitating the universal two-child policy. However, limited studies are available on risk factors for recurrence of EC in these patients. In this study, we elucidated risk factors for recurrence. Chief among them, being two metabolic features discovered to be independent risk factors. The two metabolic characteristics, insulin resistance (IR) and metabolic syndrome (MetS), substantially increased the accuracy of predicting recurrence after initial complete remission (CR).

The recurrence rate of conservative treatment varies across different studies. Chen etal. (13) reviewed 53 patients diagnosed with atypical hyperplasia or endometrioid adenocarcinoma and reported a recurrence rate of 26% in 2016. Wang etal. (14) reported that the recurrence rate ranged from 21.1 to 36.4% for patients whose treatment duration lasted from less than 6 months to more than 9 months.

Our study demonstrated that BMI, maintenance treatment, and histological type were all risk factors for recurrence and CR time after recurrence (RCR time). What’s more, we also found that diabetes was related to the length of RCR time. Body mass index (BMI), a measure of obesity and early-life obesity, has been associated with a moderately increased risk of EC later in life (15). In a randomized controlled trial, intervention weight loss for obese women could improve the survival of many cancers, including EC (16). However, the exact relationship between obesity and recurrence still remained elusive for AEH patients. Our findings were in according with this former research, in that a higher BMI correlated with worse therapeutic effects and a higher recurrence in AEH and EC patients (17). The underlying mechanisms linking obesity to relapse are still a matter of debate, but metabolic syndrome and insulin resistance/modification levels of adipocytokines appear to be of great importance.

One study found that maintenance treatment was a protective factor against relapse and RCR time. However, another study revealed that the treatment duration had no relationship with the recurrence rate (3). We concluded that maintenance treatment matters but not treatment duration for the recurrence of conservative therapy in AEH or G1EA patients.

The relationship between endometrial histological type and the prognosis of EC patients is well studied, and some of the studies stratified the patients according to the histological type (18, 19). One study concentrated on the prognostic factors for CR and found that atypical hyperplasia was easier to achieve CR (20). AEH or a lower grade was also a positive factor for a successful pregnancy and preventing recurrence (21). There are many cellular biomarkers in essential roles could be the potential mechanism underlying recurrence and lengthened RCR time, standouts include p53, p16, DNA mismatch repair proteins, PTEN, and ARID1A (22). AEH and early EC patients with diabetes, compared to those without, had worse patient characteristics, such as higher FIGO stage, similar recurrence rates, and worse overall survival (23). Our study supported this conclusion. In our analysis, patients with diabetes were more likely to experience longer CR time after recurrence.

Studies had demonstrated that IR played a vital role in the genesis and progression of many types of cancer, especially EC (24). IR and being overweight were also associated with longer therapeutic duration in EAH patients undergoing fertility-preserving treatment (25). However, the role of IR in the recurrence of AEH and early EC in patients undergoing fertility-preserving treatment remained elusive. Our study demonstrated that IR was an independent risk factor for recurrence in AEH and G1EA patients. Patients with IR needed a longer time to achieve CR after relapse compared with those without. IR was an essential risk factor for EC and may even be a possible mechanism involved in the development of EC (26). The level of insulin in patients with IR is higher than that in normal patients. It is plausible that extra insulin could bind to insulin receptors in endometrial cells promoting cancer cell proliferation, inhibiting apoptosis, and inducing angiogenesis, which in turn leads to the occurrence of EC (27). Additionally, insulin is involved in tumor development by directly or indirectly affecting endogenous estrogen metabolism and promoting the expression of endometrial estrogen receptor (ER), which in turn enhances the function of estrogen in the tumorigenesis of EC (28). It has been demonstrated that continued insulin increases the proliferation of endometrial cells under the effects of estrogen, thereby increasing the incidence of EC (28).

Another vital independent risk factor for recurrence and extended RCR time was metabolic syndrome. MetS had become one of the major worldwide public-health challenges and has been highlighted as a risk factor in several tumors, especially in EC (29). In recent years, epidemiological and clinical studies have found that MetS associated with metabolic diseases was closely related to the incidence of EC. Though there were few studies concentrating on MetS and fertility-sparing treatment in AEH and early EC patients, a great many investigations have focused on the use of metformin, an anti-MetS drug, for AEH or early EC patients with conservative therapy. Most young patients with AEH and EC who undergo fertility-sparing treatment have a background of obesity, IR and abnormal glucose tolerance complicated with polycystic ovary syndrome. Metformin had been used to counteract with these metabolic syndromes and has been attracting more attention in the field of cancer research (30). Metformin is an insulin sensitizer and has been widely investigated to treat various malignant diseases adjunctively. It has been demonstrated that metformin is an effective fertility-sparing treatment, as seen through reduction in the relapse rate after MPA therapy, particularly in obese patients (31). In the treatment of AEH and early EC, if metformin was combined with MPA, a higher early CR rate was induced compared with MPA alone.

The fact that both IR and MetS had been negatively associated with recurrence and the following CR time in progestin-based fertility-sparing treatment duration in AEH or early EC patients, indicating that IR and MetS could play synergistic roles in counteracting progestin function and compromise its therapeutic effects. Patients with both complications had the worst prognosis of fertility-preserving patients.

We hypothesized that since both IR and MetS patients have higher levels of insulin, this might induce excessive production of circulative estrogen (32). MetS is often characterized by IR, which some have suggested as a major underpinning link between physical inactivity and MetS. A possible mechanism of synergy could be that estrogen and insulin-like growth factor-1 (IGF-1) can synergistically promote the development of tumors by activating the MAPK and the AKT signaling pathways (33). Other studies have found that estrogen can bind to IGF-1R and exert non-genetic transcriptional effects through the Ras/MAPK signaling pathway and that the Ras/MAPK pathway could lead to the cancer recurrence (34, 35).

To our knowledge, this is the first study to explore the association between metabolic features and fertility-sparing treatment for AEH and early EC patients. However, there are limitations in this study. First of all, this is a single-center retrospective analysis and the number of the patients is relatively small. Moreover, endometrial tissue is collected by D&C, and this method is not as accurate as diagnostic hysteroscopy. Besides, another possible treatment of AHE or early EC also consisted of hysteroscopic removal and subsequent medical therapy. However, fertility-preserving treatment is limited by age, cancer stage, and patient desire, so it is difficult to include a large number of patients from a single institution. Therefore, a multi-center and large-population study will be needed to prove our conclusions.

## Conclusion

In conclusion, this study demonstrates that the risk factors for the recurrence and RCR time includes BMI, IR, MetS, maintenance duration, and histological type in AEH and early EC patients. Among these factors, IR and MetS are two independent risk factors which could significantly increase the accuracy of predicting recurrence in patients undergoing fertility-sparing treatment. IR and MetS may play a synergistic role in counteracting progestin function during treatment.

## Data Availability Statement

The raw data supporting the conclusions of this article will be made available by the authors, without undue reservation.

## Ethics Statement

This study was approved by the Ethics Committees of Peking University People’s Hospital (No. 2020PHB063-01).

## Author Contributions

XL and YF conceived and designed the experiments. RZ and YW collected the data. LT and JiaqW analyzed the data. XL wrote the paper. JianW and YF reviewed the draft. All authors contributed to the article and approved the submitted version.

## Funding

This study is supported by the Peking University Medicine Fund of Fostering Young Scholars’ Scientific & Technological Innovation (Grant No. BMU2021PYB012), the National Natural Science Foundation of China (Grant No. 81874108), and National Key Technology R&D Program of China (Grant No. 2019YFC1005200 and 2019YFC1005201).

## Conflict of Interest

The authors declare that the research was conducted in the absence of any commercial or financial relationships that could be construed as a potential conflict of interest.

## Publisher’s Note

All claims expressed in this article are solely those of the authors and do not necessarily represent those of their affiliated organizations, or those of the publisher, the editors and the reviewers. Any product that may be evaluated in this article, or claim that may be made by its manufacturer, is not guaranteed or endorsed by the publisher.

## Supplementary Material

Supplementary Figure 1 Recurrence-free survival in different **(A)** insulin resistance (IR); **(B)** metabolic syndrome (MetS); **(C)** different IR and MetS groups.
