# Supplementary figures and images for: Insulin Resistance and Metabolic Syndrome Increase the Risk of Relapse For Fertility Preserving Treatment in Atypical Endometrial Hyperplasia and Early Endometrial Cancer Patients
Source: Front Oncol. 2021 Nov 30;11:744689. doi: 10.3389/fonc.2021.744689 (PMC8670892; doi:10.3389/fonc.2021.744689)

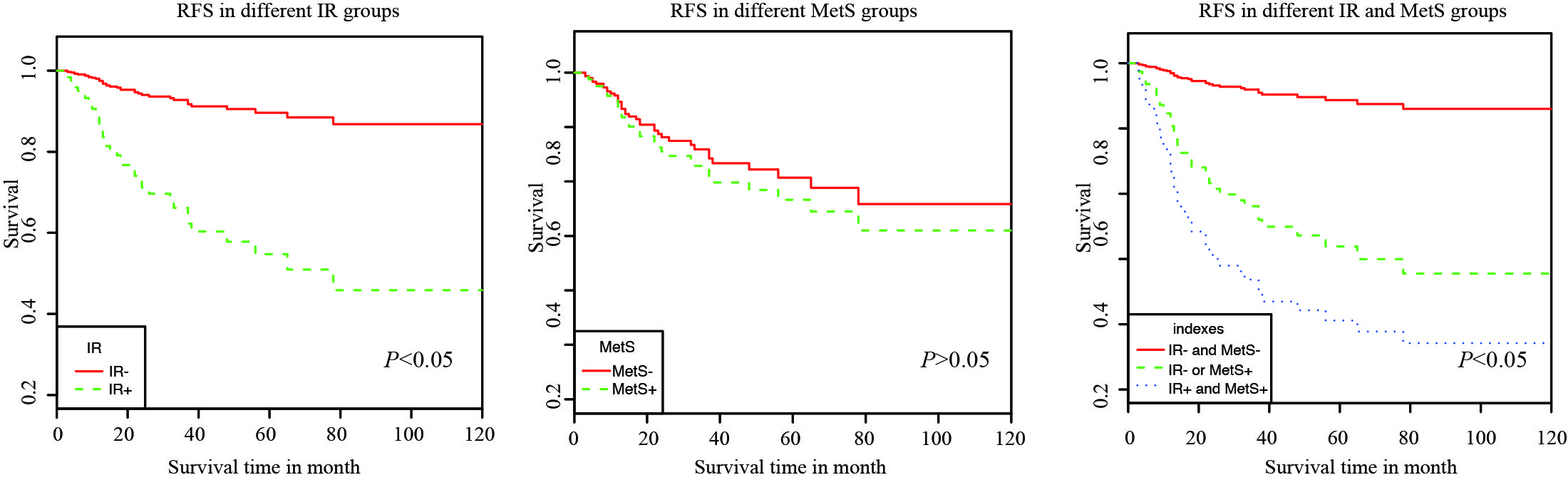

Supplement: Supplementary Figure 1 — Recurrence-free survival in different (A) insulin resistance (IR); (B) metabolic syndrome (MetS); (C) different IR and MetS groups. [file Image_1.jpeg]
