# Supplementary material for: Insulin Resistance and Metabolic Syndrome Increase the Risk of Relapse For Fertility Preserving Treatment in Atypical Endometrial Hyperplasia and Early Endometrial Cancer Patients
Source: Front Oncol. 2021 Nov 30;11:744689. doi: 10.3389/fonc.2021.744689 (PMC8670892; doi:10.3389/fonc.2021.744689)
Supplement: Supplementary file 2 [file Table_1.docx]

Table S1: Performance of the clinical model and metabolic model in predicting recurrence in the fertility-sparing treatment

| Test | Clinical Model | Metabolic Model | *P*(compare) |
| --- | --- | --- | --- |
| ROC area(AUC) | 0.7359 | 0.8183 | 0.0347 |
| 95%CI low | 0.6383 | 0.7402 |  |
| 95%CI upper | 0.8336 | 0.8964 |  |
| Accuracy | 0.6577 | 0.7658 |  |
| Specificity | 0.6301 | 0.7945 |  |
| Sensitivity | 0.7105 | 0.7105 |  |
| Positive-LR | 1.9211 | 3.4579 |  |
| Negative-LR | 0.4594 | 0.3643 |  |
| Positive-PV | 0.5000 | 0.6429 |  |
| Negative-PV | 0.8010 | 0.8406 |  |

ROC, receiver operating characteristic; AUC, area under the ROC curve; CI, confidence interval; LR, likelihood ratio; PV, predictive value.
